# Neutron structures of *Leishmania mexicana* triosephosphate isomerase in complex with reaction-intermediate mimics shed light on the proton-shuttling steps

**DOI:** 10.1107/S2052252521004619

**Published:** 2021-06-03

**Authors:** Vinardas Kelpšas, Octav Caldararu, Matthew P. Blakeley, Nicolas Coquelle, Rikkert K. Wierenga, Ulf Ryde, Claes von Wachenfeldt, Esko Oksanen

**Affiliations:** a Department of Biology, Lund University, Sölvegatan 35, 223 62 Lund, Sweden; b Department of Chemistry, Lund University, 221 00 Lund, Sweden; c Large-Scale Structures Group, Institut Laue–Langevin, 71 Avenue des Martyrs, 38042 Grenoble, France; d Faculty of Biochemistry and Molecular Medicine, University of Oulu, Pentti Kaiteran katu 1, 90570 Oulu, Finland; e European Spallation Source Consortium ESS ERIC, Odarslövsvägen 113, 224 84 Lund, Sweden

**Keywords:** triosephosphate isomerase, neutron diffraction, isomerization, quantum refinement, QM/MM, neutron crystallography, refinement, enzyme mechanisms, structural biology

## Abstract

Triosephosphate isomerase (TIM) is a key enzyme in glycolysis that catalyses the interconversion of glyceraldehyde 3-phosphate and dihydroxy­acetone phosphate. This simple reaction involves the shuttling of protons mediated by protolysable side chains. The catalytic power of TIM is thought to stem from its ability to facilitate the deprotonation of a carbon next to a carbonyl group to generate an enediolate intermediate. The enediolate intermediate is believed to be mimicked by the inhibitor 2-phosphoglycolate (PGA) and the subsequent enediol intermediate by phosphoglycolohydroxamate (PGH). Here, neutron structures of *Leishmania mexicana* TIM have been determined with both inhibitors, and joint neutron/X-ray refinement followed by quantum refinement has been performed. The structures show that in the PGA complex the postulated general base Glu167 is protonated, while in the PGH complex it remains deprotonated. The deuteron is clearly localized on Glu167 in the PGA–TIM structure, suggesting an asymmetric hydrogen bond instead of a low-barrier hydrogen bond. The full picture of the active-site protonation states allowed an investigation of the reaction mechanism using density-functional theory calculations.

## Abstract 2

The high-resolution neutron structure of triosephosphate isomerase was determined in complex with two inhibitors that mimic intermediate states in the reaction mechanism. This shows clear protonation states of the catalytic residues, which allow a computational investigation of the disputed reaction mechanism.

## 1. Introduction   

Triosephosphate isomerase (TIM; EC 5.3.1.1) plays a central role in glycolysis by interconverting dihydroxyacetone phosphate (DHAP) and glyceraldehyde 3-phosphate (GAP). Both substrates are produced in the reaction catalyzed by fructose-1,6-bisphosphate aldolase (EC 4.1.2.13). GAP is used in downstream reactions to produce substrates for the tri-carboxylic acid cycle.

The kinetic mechanism of TIM has been very well studied, and TIM is often called a catalytically perfect enzyme because of the high rate and high catalytic efficiency of the GAP-to-DHAP reaction (Zhai *et al.*, 2015 ▸). The *k*_cat_/*K*_m_ value of *Saccharomyces cerevisiae* TIM is 8.9 × 10^6^ *M* ^–1^ s^–1^ for the GAP-to-DHAP reaction, which is close to the diffusion-controlled rate limit (Albery & Knowles, 1977 ▸). The free-energy profile reveals that the TIM-catalyzed reaction is 10^9^ times faster compared with general base catalysis in solution (Albery & Knowles, 1976 ▸). *k*_cat_ for the DHAP-to-GAP reaction is 860 s^–1^, while *k*_cat_ for the reverse reaction is approximately ten times larger (Zhai *et al.*, 2015 ▸).

There are several conserved active-site residues in TIM; here, we use the numbering of the *Leishmania mexicana* enzyme (*Lm*TIM). The catalytic roles of Glu167, Asn11, Lys13 and His95 have been studied extensively. Asn11 and Lys13 provide electrostatic stabilization (Go *et al.*, 2010 ▸), and together with His95 form an oxyanion-hole-like environment for the intermediates (Wierenga *et al.*, 2010 ▸). Glu167 is thought to act as the catalytic base in the initial, rate-limiting proton-abstraction step to generate an enediolate intermediate, which is further stabilized by other residues in the active site.

**Figure 1:**
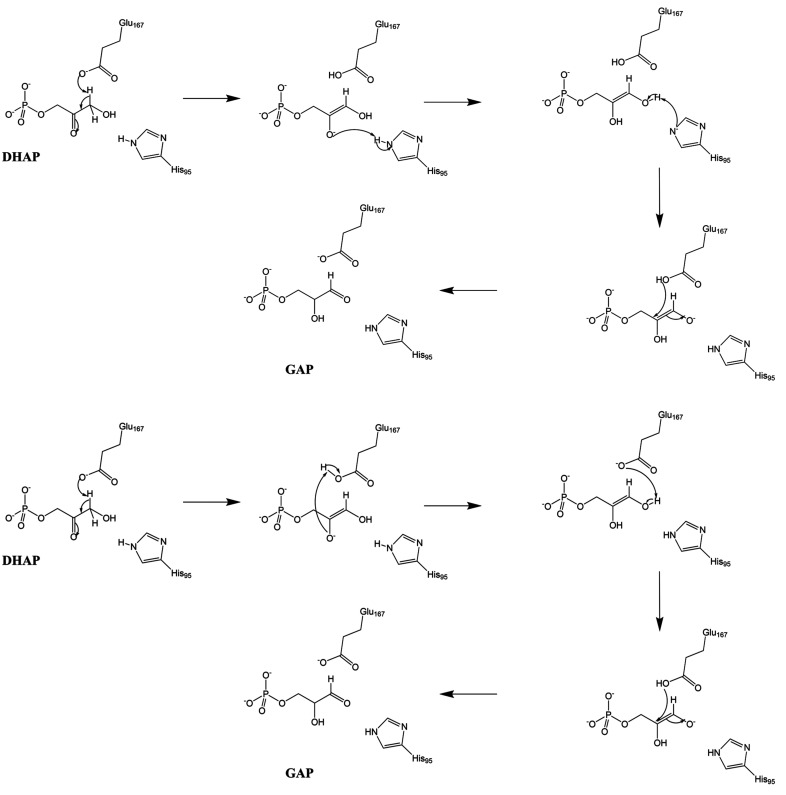
The classical (top) and criss-cross (bottom) mechanisms for the isomerization of DHAP to GAP catalyzed by TIM. The shuffle mechanism skips the enediol intermediate in the classical mechanism (not depicted).

Three alternate mechanisms for the subsequent proton-shuttling steps have been proposed (Fig. 1 ▸). In the classical mechanism, His95 donates a proton to the enediolate oxygen and then abstracts a proton from the other hydroxyl group of the enediol (Knowles, 1991 ▸). In the criss-cross mechanism, the protonated Glu167 first reprotonates the charged enediolate oxygen, and then abstracts another proton from the other hydroxyl group of the resulting enediol (Harris *et al.*, 1998 ▸). In the criss-cross mechanism, the role of His95 is solely to stabilize the negative charge through strong hydrogen bonds. Another possibility is that the classical mechanism is performed in only one step, with the two protons being transferred concurrently. This would avoid the formation of an intermediate in which His95 would have a negative charge. We call this the shuffle mechanism. The isotope-exchange data (Harris *et al.*, 1998 ▸) cannot unambiguously distinguish between the mechanisms and are consistent with both mechanisms contributing simultaneously.

**Figure 2:**
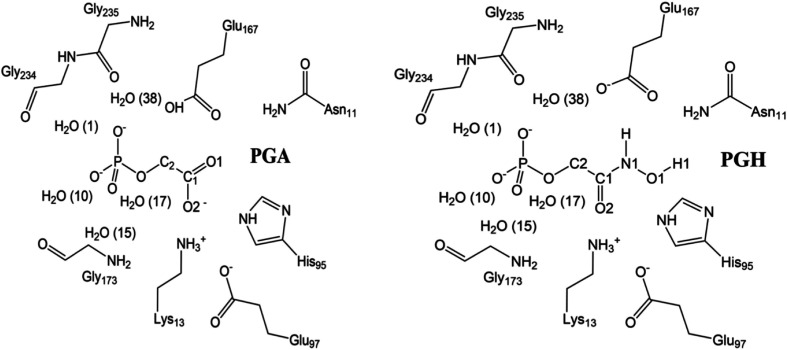
Scheme of the active site of TIM with the inhibitors PGA (left) and PGH (right), also showing the atom numbering of the inhibitor and the waters. The atoms shown were used as the quantum system for the QM/MM calculations. The overall charge of PGA and PGH is −3 and −2, respectively.

TIM is a very fast enzyme, so any intermediates are short-lived compared with the turnover rate (Jogl *et al.*, 2003 ▸). Therefore, mechanism-based inhibitors that mimic the enediolate and enediol intermediates need to be used for structural studies. 2-Phosphoglycolate (PGA; Fig. 2 ▸) has been considered to be a mimic of the enediolate intermediate (Wolfenden, 1969 ▸) and has a *K*_i_ of 26 µ*M* (Schliebs *et al.*, 1996 ▸), having a much higher affinity than DHAP, which has a *K*_m_ of 300 µ*M* for *Lm*TIM (Kohl *et al.*, 1994 ▸). It has been shown that a residue becomes protonated upon the binding of PGA to TIM, and it was assumed that this was Glu167 (Campbell *et al.*, 1979 ▸).

Elegant studies by the John Richard group on the protonation state of the Glu167 side chain show that the p*K* changes from ∼4 to ∼10 when forming a complex with PGA, mimicking the protonation event of this glutamate upon substrate binding (Malabanan *et al.*, 2013 ▸). Another inhibitor, phosphoglycolohydroxamate (PGH; Fig. 2 ▸; Collins, 1974 ▸), with a *K*_i_ of 8 µ*M*, has been used to mimic the uncharged enediol intermediate (Schliebs *et al.*, 1996 ▸). These molecules have been extensively used in X-ray crystallographic studies at subatomic resolution. The neutral protonation state of His95 was discernible in these structures, but the protonation states of the inhibitors and Glu167 could not be determined.

Another interesting topic in the mechanism of TIM is the possible presence of low-barrier hydrogen bonds (LBHBs). An LBHB between Glu167 and the substrate was initially thought to reduce the activation energy for the proton transfer and the formation of enediolate. An LBHB between the inhibitor PGH and Glu167 was suggested by a previous NMR study (Harris *et al.*, 1997 ▸). The atomic resolution structure of *Lm*TIM with PGA (Kursula & Wierenga, 2003 ▸) shows a hydrogen bond of 2.61 Å between the carboxylate group of the inhibitor and Glu167. Likewise, the crystal structure of the PGH complex (Alahuhta & Wierenga, 2010 ▸) shows hydrogen bonds of 2.60 and 2.69 Å between the two Glu167 carboxylate O atoms and the O and N atoms of the hydroxamate group of PGH, respectively. In both cases, it was suggested that Glu167 is protonated and therefore neutral.

However, even at subatomic resolution it is difficult to answer such hydrogen-related questions using X-ray crystallography. Neutron crystallography allows the determination of the atomic positions of H atoms in macromolecular structures even at modest resolutions (Blakeley, 2009 ▸). Neutron structures of TIM in complex with inhibitors therefore provide an unequivocal assignment of the protonation states of the residues in the active site and information about the nature of the hydrogen bonds in which these participate.

In this study, we have determined neutron crystal structures of *Lm*TIM with PGH and PGA, obtained by joint X-ray/neutron refinement, to unravel the protonation states in the active site. We have used our quantum-mechanical (QM) refinement approach (Caldararu *et al.*, 2019 ▸) to support the protonation-state assignments in the crystal structures. We have also used hybrid QM and molecular-mechanics (QM/MM) calculations to study the mechanism of the *Lm*TIM-catalyzed isomerization.

## 2. Methods   

The thermostable E65Q variant of *Lm*TIM was used in the structures reported here. The enzyme kinetic properties of E65Q and wild-type *Lm*TIM are essentially identical (Williams *et al.*, 1999 ▸). The perdeuteration and crystal growth, as well as neutron and X-ray data collection and processing, have been described previously (Kelpšas *et al.*, 2019 ▸). In addition, we determined the X-ray structure of the PGA complex (PDB entry 7abx, Supplementary Table S2) at 100 K to exclude any heavy-atom changes due to perdeuteration. The neutron data were collected using the LADI-III instrument (Blakeley *et al.*, 2010 ▸) at the Institut Laue–Langevin, Grenoble, France. Data-collection statistics are reported in Supplementary Table S1. The column names for the reflection data are explained in the supporting information.

Conventional crystallographic refinement was performed with *Phenix* 1.14 (Liebschner *et al.*, 2019 ▸), using the *phenix.refine* module, as described previously (Kelpšas *et al.*, 2019 ▸). OMIT maps were generated with the *phenix.polder* utility by excluding the D atoms and the solvent mask around them from map calculation.

Quantum refinement is standard crystallographic refinement combined with QM calculations for a small but interesting part of the protein (Ryde & Nilsson, 2003 ▸; Ryde *et al.*, 2002 ▸). As crystallographic refinement uses geometry restraints akin to a molecular-mechanics (MM) force field, a QM/MM formalism can be applied, replacing the geometry restraints for part of the protein with QM calculations. This yields the following target function:

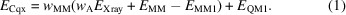




The weight term *w*_MM_ is needed to scale the energies from crystallographic geometry restraints, which are not energy-based, to the QM energies. Previous studies have shown that a factor of 1/3 for this weight is typical (Nilsson & Ryde, 2004 ▸). The MM energy of the quantum system (*E*_MM1_) needs to be subtracted from the total MM energy (*E*_MM_) to avoid double counting. The *w*_A_ term determines the relative importance of the X-ray and MM or QM terms. This energy function can easily be extended to joint X-ray/neutron quantum refinement by simply adding a neutron-data term (Caldararu *et al.*, 2019 ▸),

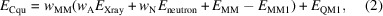

where *w*_N_ is another weight between the neutron data and the other terms.

Joint X-ray/neutron quantum refinement has been implemented in the *ComQum-U* software (Caldararu *et al.*, 2019 ▸), which combines the neutron version of the *Crystallography & NMR System* (*nCNS*; Adams *et al.*, 2009 ▸) with the QM software *TURBOMOLE* (Furche *et al.*, 2014 ▸). *ComQum-U* was used to perform all quantum refinements. All of the QM calculations within quantum refinements were performed at the TPSS/def2-SV(P) level, sped up with the resolution-of-identity approximation (Sierka *et al.*, 2003 ▸). As all of the H-atom positions are known from the neutron structures, atoms outside the quantum system were represented by partial point charges. Thereby, the polarization of the QM system by the surroundings is included in a self-consistent manner.

Various values of the *w*_A_ and *w*_N_ weights were tested as described previously (Caldararu *et al.*, 2019 ▸), and a weight of 3 for both the neutron and the X-ray data was chosen for all quantum-refinement calculations.

QM/MM calculations were performed with the *ComQum* software (Ryde, 1996 ▸; Ryde & Olsson, 2001 ▸). In this approach, the protein and solvent are split into two subsystems. System 1 (the QM region) was relaxed by QM methods, whereas system 2 contained the remaining part of the protein and the solvent. System 2 was kept fixed at the coordinates obtained from the quantum refinements.

When there is a bond between systems 1 and 2 (a junction), the hydrogen link-atom approach was employed. The QM system was capped with H atoms [hydrogen link (HL) atoms], the positions of which are linearly related to the corresponding C atoms [carbon link (CL) atoms] in the full system (Reuter *et al.*, 2000 ▸). All atoms were included in the point-charge model, except for the CL atoms (Hu & Ryde, 2011 ▸).

The total QM/MM energy in *ComQum* was calculated as

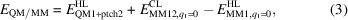

where 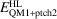 is the QM energy of the QM system truncated by HL atoms and embedded in the set of point charges modelling system 2 (but excluding the self-energy of the point charges). 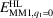 is the MM energy of the QM system, still truncated by HL atoms, but without any electrostatic interactions. Finally, 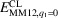 is the classical energy of all atoms in the system with CL atoms and with the charges of the QM region set to zero (to avoid double counting of the electrostatic interactions). Thus, *ComQum* employs a subtractive scheme with electrostatic embedding and van der Waals link-atom corrections (Cao & Ryde, 2018 ▸).

The QM/MM geometry optimizations were performed using the TPSS method and either the def2-SV(P) or the def2-TZVPD basis sets, and also at the B3LYP/def2-TZVPD level of theory. For both functionals, empirical dispersion corrections were included with the DFT-D3 approach (Grimme *et al.*, 2010 ▸) and Becke–Johnson damping (Grimme *et al.*, 2011 ▸).

The quantum system for all quantum refinements and QM/MM calculations consisted of the substrate or inhibitor (DHAP, PGA or PGH), as well as the side chains of Asn11, Lys13, His95, Glu97, Glu167, Gly173, Gly234 and Gly235. The quantum system also included five water molecules: Wat1, Wat10, Wat15, Wat17 and Wat38 according to the numbering in the PGA–TIM crystal structure. The quantum system is shown in Fig. 2 ▸.

The coordinates and structure factors have been deposited in the Protein Data Bank with accession codes 7abx (PGA–TIM, cryo X-ray), 7az3 (PGA–TIM), 7az4 (QM-refined PGA–TIM), 7az9 (PGH–TIM) and 7aza (QM-refined PGH–TIM). All figures showing 3D structures were generated with the *PyMOL* Molecular Graphics System (version 1.8; Schrödinger).

## 3. Results   

### 3.1. Refinement of the PGA complex   

The X-ray refinement of perdeuterated *Lm*TIM in complex with the inhibitor PGA (PDB entry 7abx) at 1.19 Å resolution at 100 K shows a structure of the active site that is similar to that in the 0.82 Å resolution structure of the PGH complex at 100 K (PDB entry 2vxn) reported by Alahuhta & Wierenga (2010 ▸). The all-atom r.m.s.d. of the two structures is 0.15 Å, whereas the r.m.s.d. of the residues in the active site (excluding D atoms and the alternative conformations of PGH and PGA found in PDB entry 2vxn) is 0.20 Å. The differences between the room-temperature structure (Kelpšas *et al.*, 2019 ▸) and the 1.19 Å resolution structure of the perdeuterated protein at 100 K are also small, with an all-atom r.m.s.d. of 0.12 Å. Moreover, none of the residues in the active site show any significant geometric changes between the room-temperature and 100 K structures.

**Figure 3:**
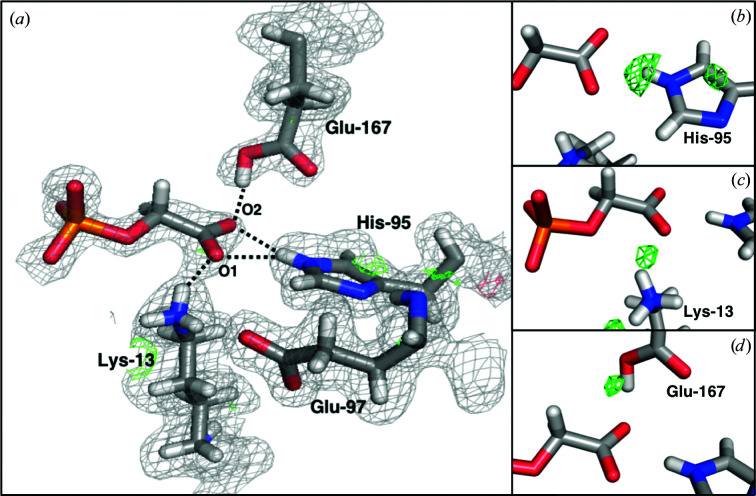
(*a*) Joint X-ray/neutron-refined structure of PGA–TIM with the 2*mF*_o_ − *DF*_c_ nuclear scattering-length density map contoured at 1.0σ and the *mF*_o_ − *DF*_c_ nuclear scattering-length difference density maps contoured at 3.0σ (green) and −3.0σ (red). (*b*–*d*) Nuclear scattering-length OMIT maps at 3.0σ of the D atoms of three important residues in the PGA–TIM active site: (*b*) His95, (*c*) Lys13 and (*d*) Glu167.

Adding the 1.7 Å resolution neutron data to the refinement process (through joint X-ray/neutron refinement) reveals unambiguous protonation states of some residues. His95 has a single deuteron at the N^ɛ^ atom (Fig. 3 ▸), which is in accordance with the H atom observed in the atomic resolution structure PDB entry 2vxn and is confirmed by the OMIT map without the N^ɛ^ deuterium [Fig. 3 ▸(*b*)]. It forms a bifurcated hydrogen bond to the two O atoms of the carboxyl group of PGA (H–O distances of 2.06 and 2.41 Å). The latter group is deprotonated and hence negatively charged. The phosphate group of PGA is fully deprotonated because it forms hydrogen bonds as the acceptor to several water molecules and the backbone amides of Gly173, Gly234 and Gly235. We also conclude that Lys13 is deuterated and hence positively charged, as an OMIT map with a neutral lysine gives rise to clear positive difference density in the nuclear map. The Lys13 residue forms a 2.0 Å hydrogen bond to the O1 atom of PGA, with the nuclear density between these two atoms appearing continuous at the 1.0σ contour level in the 2*mF*_o_ − *DF*_c_ nuclear scattering-length density map. Glu167 is also deuterated, forming a short hydrogen bond to the O2 atom of PGA with a hydrogen–acceptor distance of 1.6 Å. The Glu167 deuteron density is less pronounced than for the His95 and Lys13 residues, but OMIT maps also confirm that this is the observed state of the residue [Fig. 3 ▸(*d*)]. The neutral state of Glu167 is also consistent with the previously reported full-matrix least-squares refinement of the subatomic resolution X-ray data at 100 K (Alahuhta & Wierenga, 2010 ▸). As expected, nuclear scattering-length density maps also reveal that the two alkyl H atoms of PGA have not exchanged to deuteriums and were therefore modelled as H instead of D in all refinements. Nuclear density map details for Glu167 and His95 are provided for clarification (Supplementary Fig. S1), along with the coordinates and nuclear scattering-length density maps for the active site, including the surrounding residues (Supplementary Fig. S2).

**Figure 4:**
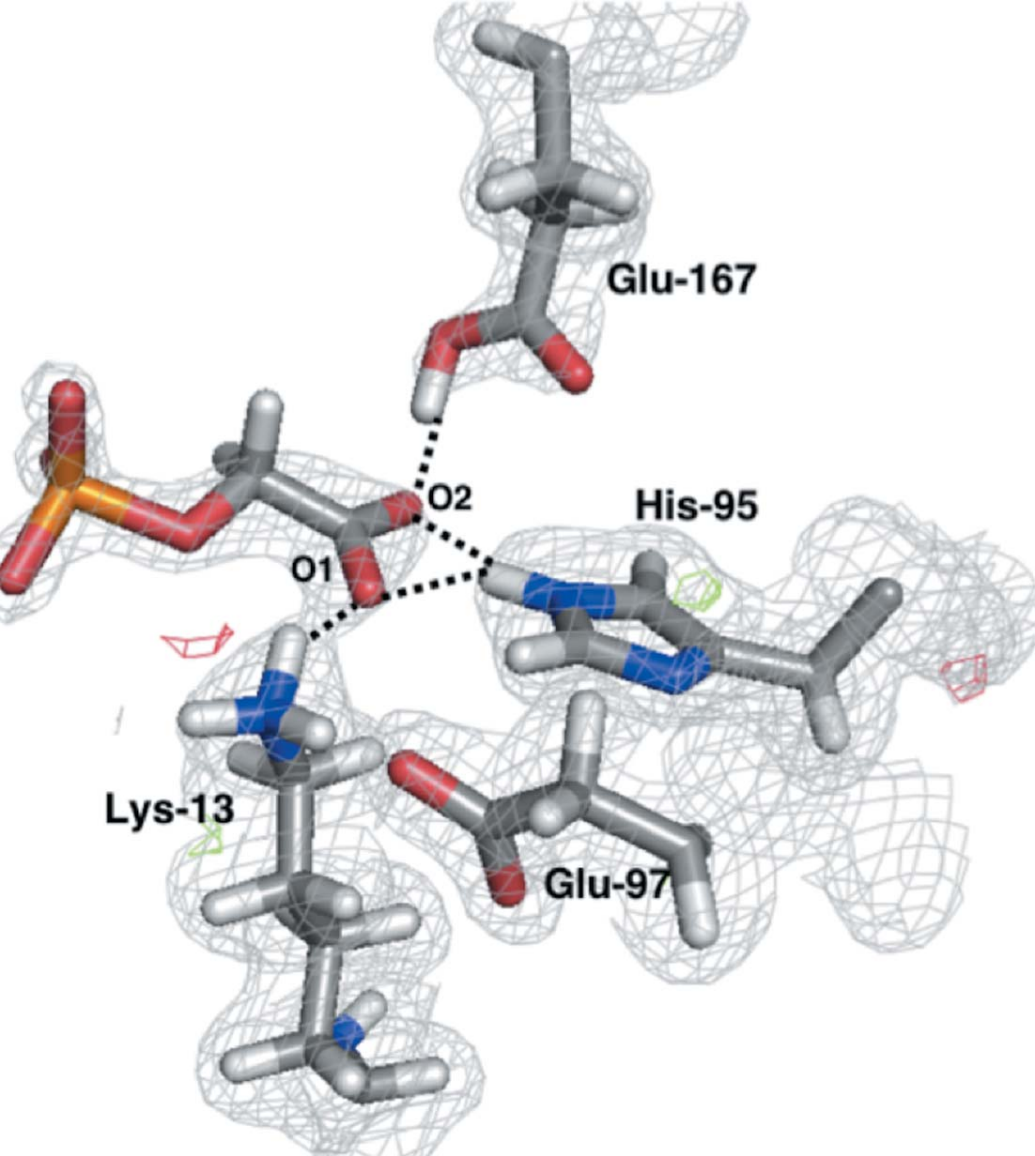
Quantum-refined joint X-ray/neutron structure of PGA–TIM with the 2*mF*_o_ − *DF*_c_ nuclear scattering-length density map contoured at 1.0σ and the *mF*_o_ − *DF*_c_ nuclear scattering-length difference density maps contoured at 3.0σ (green) and −3.0σ (red).

To validate the deuteration states of important residues and to improve the description of the hydrogen-bond network in the active site, we also performed joint X-ray/neutron quantum refinement of the PGA–TIM complex (Caldararu *et al.*, 2019 ▸). We tested three cases: (i) the deuteration state of the traditionally refined structure (both Lys13 and Glu167 protonated), (ii) Lys13 neutral, with the D atom on the O1 atom of PGA, and (iii) Glu167 negatively charged, with the D atom on the O2 atom of PGA. In all cases, the optimized structure led back to the initial traditionally refined structure with Lys13 and Glu167 protonated, showing that these protonation states are the most chemically reasonable, which is in accordance with the results obtained from the diffraction data alone. As seen in Fig. 4 ▸, the quantum-refined structure of PGA–TIM and the nuclear density maps look almost identical to the traditional joint X-ray/neutron-refined structure and maps. This is a strong indication that the deuteration-state assignments are correct, in particular that of Glu167, for which the nuclear density by itself is not completely unambiguous. The quantum refinement also shortened all hydrogen bonds between PGA and the protein residues, which is expected as in standard refinement we did not restrain the hydrogen-bond distances. Quantum refinement, however, allows the use of this information in an unbiased way and hence produces a slightly better hydrogen-bonding geometry. His95 still forms a bifurcated hydrogen bond, but with H–O distances of 1.81 and 2.37 Å, respectively. The Lys13–O1 hydrogen-bonding distance was reduced to 1.68 Å, whereas the Glu167–O2 distance was reduced to 1.51 Å.

Although the latter very short hydrogen bond might point towards an LBHB, neither the experimental nuclear density nor the quantum-chemical calculations suggest the presence of an LBHB here, but rather a strong asymmetric hydrogen bond. Refinement details of the refined and quantum-refined PGA–TIM structures are shown in Table 1 ▸.

### 3.2. Refinement of the PGH complex   

The 1.8 Å resolution neutron crystal structure of PGH–TIM shows an almost identical overall structure to the PGA–TIM structure, with an all-atom r.m.s.d. of 0.09 Å. The r.m.s.d. between active-site residues is also only 0.15 Å, with the O1 atom of PGA superposing on the N atom of PGH.

**Figure 5:**
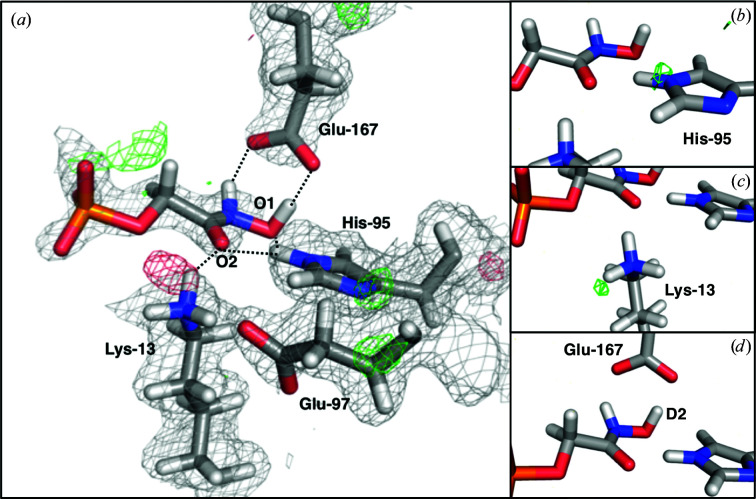
Joint X-ray/neutron-refined structure of PGH–TIM with the 2*mF*_o_ − *DF*_c_ nuclear scattering-length density map contoured at 1.0σ and the *mF*_o_ − *DF*_c_ nuclear scattering-length difference density maps contoured at 3.0σ (green) and −3.0σ (red). (*b*–*d*) Nuclear scattering-length OMIT maps at 3.0σ of the D atoms of three important residues in the PGH–TIM active site: (*b*) His95, (*c*) Lys13 and (*d*) PGH (D2).

The assignment of protons from nuclear scattering-length density is less clear than in the case of PGA–TIM. The N^ɛ^ atom of His95 is deuterated, which is also obvious from the His95 OMIT map [Fig. 5 ▸(*b*)], but the deuteration states of Lys13, Glu167 and the PGH inhibitor itself are not clear from the maps derived from traditional joint X-ray/neutron crystallographic refinement alone [Fig. 5 ▸(*a*)]. Indeed, even the OMIT maps of Lys13 and PGH are ambiguous as to the presence or absence of these deuteriums [Figs. 5 ▸(*c*) and 5 ▸(*d*)].

No nuclear density was observed around the hydroxylamine deuteron and oxygen of PGH (O1 and H1 in Fig. 2 ▸). Furthermore, modelling H1 as a deuterium gave rise to negative difference density, similar to that observed on the aliphatic PGH protons if modelled as deuteriums. Thus, either the H1 atom has not exchanged or the OH group is rotationally disordered and is not observable in the PGH–TIM crystal structure. The lower completeness of the neutron diffraction data (Supplementary Table S1) could also have affected the nuclear density map. Moreover, we cannot be certain whether PGH or the Glu167 residue is negatively charged, but the previously reported full-matrix least-squares refinement of subatomic resolution X-ray data at 100 K (Alahuhta & Wierenga, 2010 ▸) suggests Glu167 to be deprotonated. The Lys13 deuteron involved in a hydrogen bond to the ketone oxygen of PGH also gives rise to negative difference density at 3.0σ; thus, the deuteration state of this residue is not fully clear based on standard crystallographic refinement.

**Figure 6:**
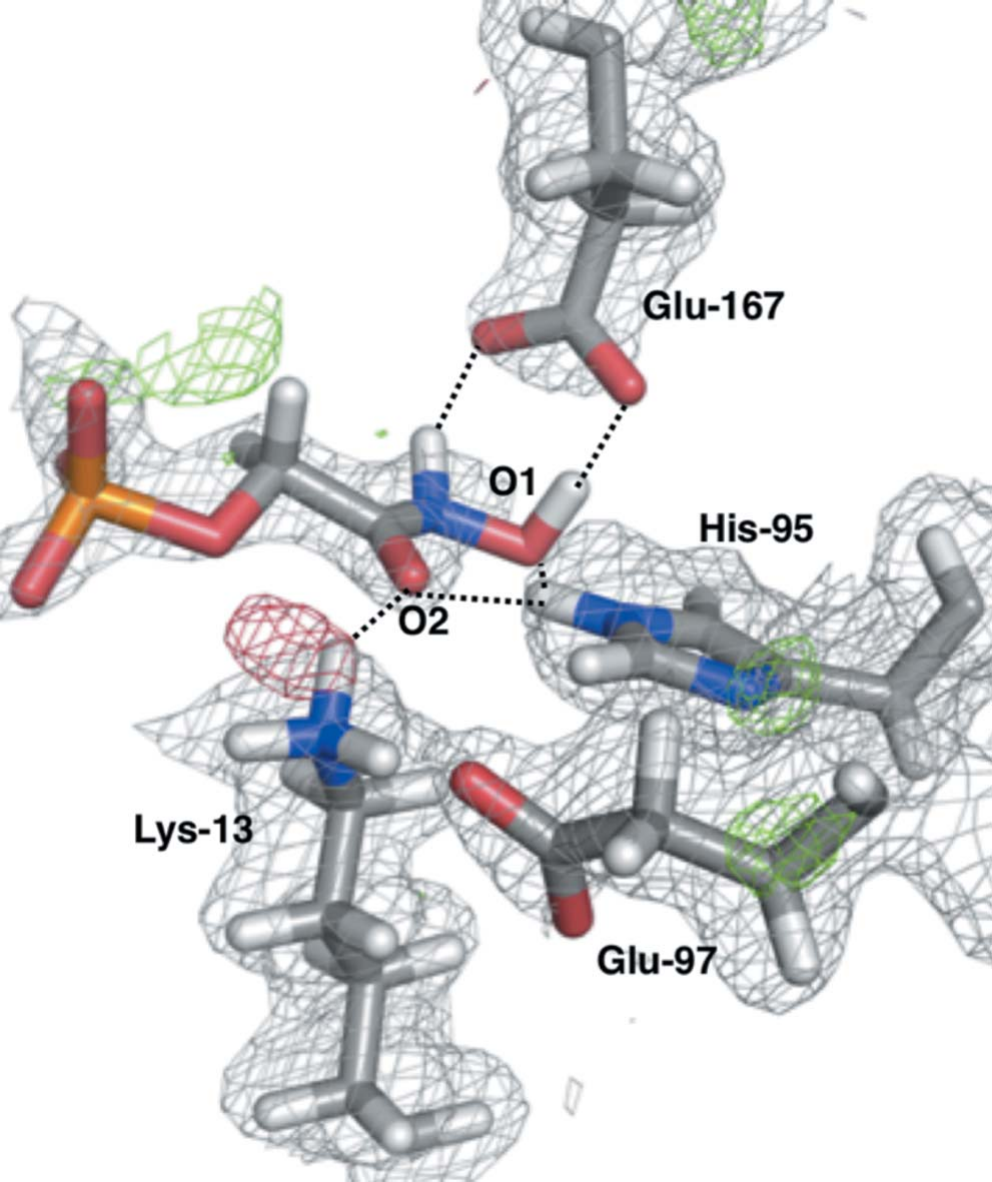
Quantum-refined joint X-ray/neutron structure of PGH–TIM with the 2*mF* − *DF*_c_ nuclear scattering-length density map contoured at 1.0σ and the *mF*_o_ − *DF*_c_ nuclear scattering-length difference density maps contoured at 3.0σ (green) and −3.0σ (red).

To elucidate the deuteration states of the active-site residues, we performed quantum refinement of the PGH–TIM crystal structure, testing all possible deuteration states of the Lys13 and Glu167 residues and the PGH ligand, but keeping the His95 residue deuterated at N^ɛ^, as concluded from the traditionally refined structure. In all cases, quantum refinement led to a structure with Lys13 deuterated (positively charged), Glu167 nondeuterated (negatively charged) and PGH in its deuterated hydroxylamine form (Fig. 6 ▸). These results show a clear structure that is both the most chemically reasonable according to QM calculations and conforms to the observed neutron and X-ray diffraction data. This indicates that the negative *mF*_o_ − *DF*_c_ density is not an indication of lysine dedeuteration and that the OH group of PGH is indeed rotationally disordered. This conclusion would not have been possible without quantum refinement, which also gave a clearer picture of the hydrogen-bond interactions in the active site. As in the PGA–TIM structure, His95 forms a bifurcated hydrogen bond to the two O atoms of PGH, with H–O distances of 1.8 and 2.0 Å, respectively. Lys13 forms a 1.8 Å hydrogen bond to the ketone O atom of PGH, while Glu167 forms a very short 1.6 Å bond to the hydroxyl atom as a donor. As the hydroxylamine oxygen is disordered even in the room-temperature X-ray structure, the neutron data unfortunately give little insight into the nature of this hydrogen bond as an LBHB. Refinement details of the refined and quantum-refined PGH–TIM structures are shown in Table 2 ▸. Coordinates and nuclear scattering-length density maps of the active site, including the surrounding residues, are shown in Supplementary Fig. S3.

### 3.3. Reaction mechanism   

The inhibitor PGA is thought to be a good mimic of the first intermediate in the DHAP-to-GAP isomerization reaction catalysed by TIM. In order to test this hypothesis and verify that the PGA–TIM structure is relevant to the enzymatic catalysis pathway, we performed computational studies of the isomerization mechanism of TIM.

All three reaction mechanisms of TIM start with the deprotonation of DHAP by Glu167, yielding an enediolate intermediate (Fig. 1 ▸). The starting structure was obtained by replacing the PGA inhibitor in the quantum-refined structure by DHAP, using the 1.2 Å resolution crystal structure of TIM in complex with DHAP (PDB entry 1ney; Jogl *et al.*, 2003 ▸) as the template. The Glu167 residue was considered to be deprotonated in the reactant state, whereas all of the other residues were assigned the protonation states observed in the refined neutron structure.

**Figure 7:**
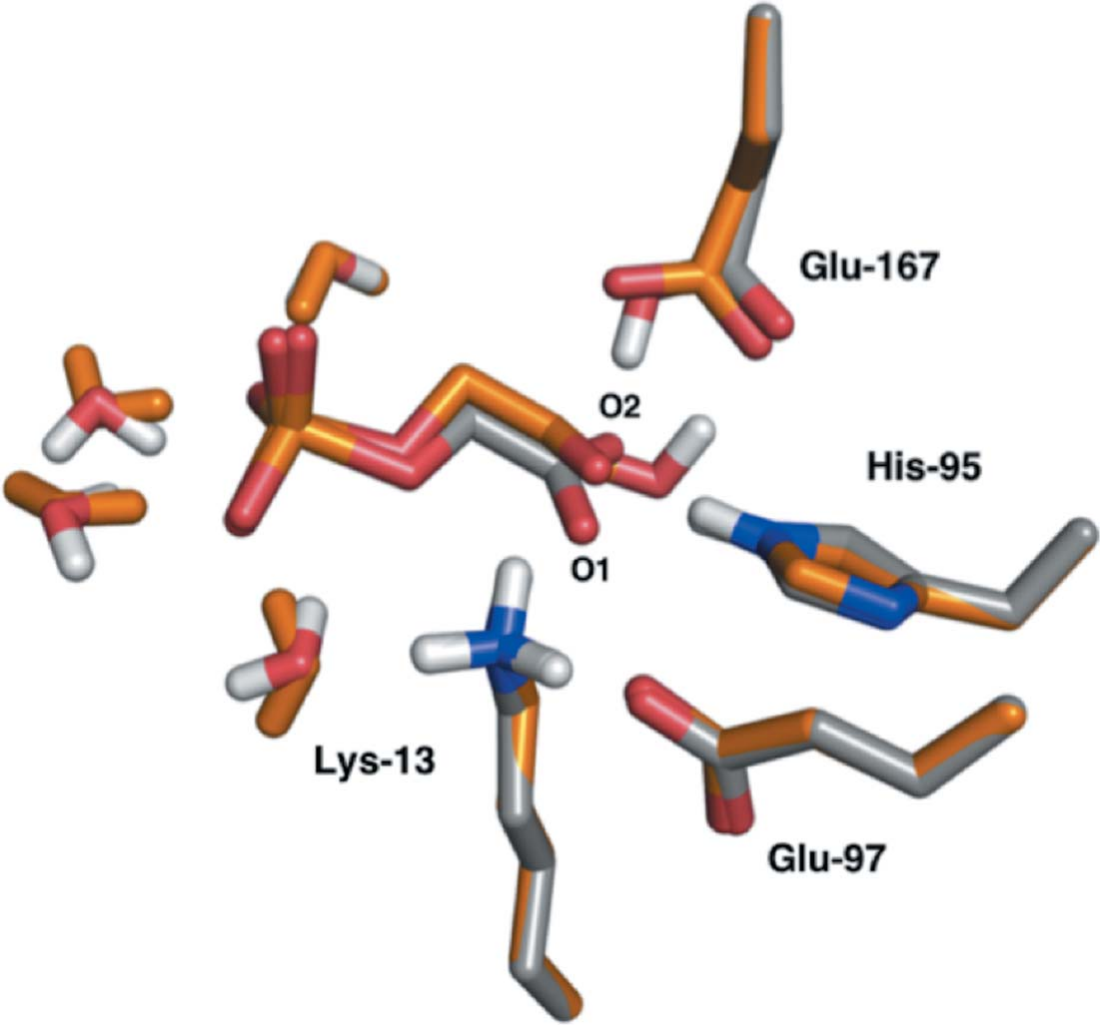
Superposition of the QM/MM-optimized structure of DHAP–TIM (orange) and the quantum-refined structure of PGA–TIM (grey).

In the QM/MM-optimized structure of DHAP–TIM (Fig. 7 ▸) there are no significant conformational changes of the residues in the active site and no movements of the water molecules that form hydrogen bonds to the DHAP ligand. The r.m.s.d. between the two active sites is 0.62 Å. The O2 atom of PGA superposes well with the C1 atom of DHAP, but the distance between Glu167 and the H atom to be abstracted from DHAP becomes 2.5 Å, which is 1 Å longer than the Glu167–O2 distance in the PGA–TIM complex. The only other difference in the active site with DHAP is the orientation of the ketone oxygen of DHAP compared with the O1 atom of PGA. Lys13 still forms a hydrogen bond to DHAP, but it is much weaker, with a hydrogen-bond distance of 2.3 Å compared with 1.6 Å in the PGA complex. This is expected as the O1 atom in PGA is charged according to the protonation states observed in the neutron structure.

The deprotonation reaction was initially attempted within the QM/MM framework at the TPSS/def2-SV(P) level of theory, but it was found to be barrierless and we were not able to find any product or transition state, so no reaction and activation energies could be calculated at this level of theory.

However, increasing the size of the basis set to def2-TZVPD made the calculation possible, revealing a very flat potential energy surface for the reaction. The deprotonation energy calculated by QM/MM was 9.4 kcal mol^−1^, whereas the activation energy of the reaction was only 9.7 kcal mol^−1^.

We then attempted the next step in the isomerization of DHAP to GAP. We considered the classical, criss-cross and shuffle mechanisms in this step. In the classical mechanism the histidine N^ɛ^ proton is transferred to the ketone oxygen of DHAP, whereas in the criss-cross mechanism Glu167 donates back the proton it extracted in the previous step to form the enediol intermediate. The reaction energy for proton transfer from His95 was 7.0 kcal mol^−1^ at the TPSS/TZVPD level of theory and the activation energy was 7.7 kcal mol^−1^. On the other hand, the results showed an exothermic step in the criss-cross mechanism, with a reaction energy of −0.8 kcal mol^−1^. The calculated activation energy for this step was 5.7 kcal mol^−1^.

In the classical mechanism, reprotonation of His95 by the hydroxide group of the enediol, forming a second enediolate intermediate, was highly exothermic at −7.9 kcal mol^−1^, with an activation energy of only 0.5 kcal mol^−1^. In the criss-cross mechanism, the same enediolate intermediate is also formed. However, the reaction energy was much lower at only −0.1 kcal mol^−1^. The activation energy for this step in the criss-cross mechanism was 6.6 kcal mol^−1^. This is slightly lower than the net activation energy in the classical mechanism (15.2 kcal mol^−1^ compared with 16.9 kcal mol^−1^ for the classical mechanism, both relative to DHAP).

In the shuffle mechanism, there is only one step between the two enediolate intermediates and the enediol intermediate is never formed, because the deprotonation and reprotonation of His95 occur concurrently. The single step of the shuffle mechanism has an exothermic reaction energy of −0.9 kcal mol^−1^ and an activation energy of 8.1 kcal mol^−1^.

**Figure 8:**
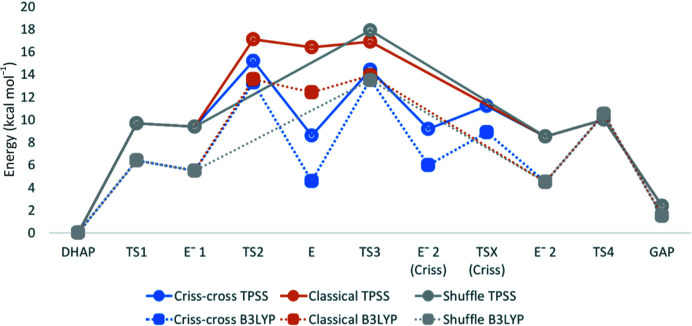
Reaction path of the TIM-catalyzed isomerization of DHAP to GAP calculated by QM/MM for three different mechanisms: criss-cross (blue), classical (orange) and shuffle (grey).

The last step of the reaction, forming GAP, is also exothermic by 6.1 kcal mol^−1^. The activation energy of this step is 1.8 kcal mol^−1^. Thus, the total QM/MM reaction energy of the isomerization of DHAP to GAP catalysed by TIM is 2.4 kcal mol^−1^. The relative energies of the reaction intermediates and transition states in all three mechanisms are shown in Table 3 ▸ and Fig. 8 ▸. From these, it can be seen that the rate-limiting barrier is lowest for the criss-cross mechanism, at 15.2 kcal mol^−1^ for the third transition state, whereas it is highest for the shuffle mechanism at 17.5 kcal mol^−1^ and is intermediate for the classical mechanism at 16.9 kcal mol^−1^ for the second transition state.

To check the reliability of the results, we also performed single-point B3LYP-D3/def2-TZVPD calculations (this method is expected to give more reliable results for such reactions involving proton transfers and no transition metals; Blomberg *et al.*, 2014 ▸) on the TPSS structures. The B3LYP energies of most of the intermediates and transition states are 3–4 kcal mol^−1^ lower than the TPSS energies. With B3LYP, the shuffle mechanism has the lowest net activation barrier, at 13.5 kcal mol^−1^ (for the third transition state), but it is only 0.4–1.1 kcal mol^−1^ lower than for the other two mechanisms.

The rate-limiting barriers for all three mechanisms, especially those obtained with B3LYP, are in good agreement with the observed *k*_cat_ for the DHAP-to-GAP reaction, 860 s^–1^, which corresponds to a barrier of 13.5 kcal mol^−1^. The small difference in the net barriers (0.4–2.3 kcal mol^−1^) and the variation with the DFT functional makes it hard to suggest that any of the three mechanisms is better than the others. Previous calculations by Cui & Karplus (2001 ▸, 2002 ▸) also suggested that both the classical and the criss-cross mechanisms contribute to the TIM isomerization reaction, with similar net activation energies (12–14 kcal mol^−1^), although they only used a QM region with the substrate and catalytic residues in calculations.

**Figure 9:**
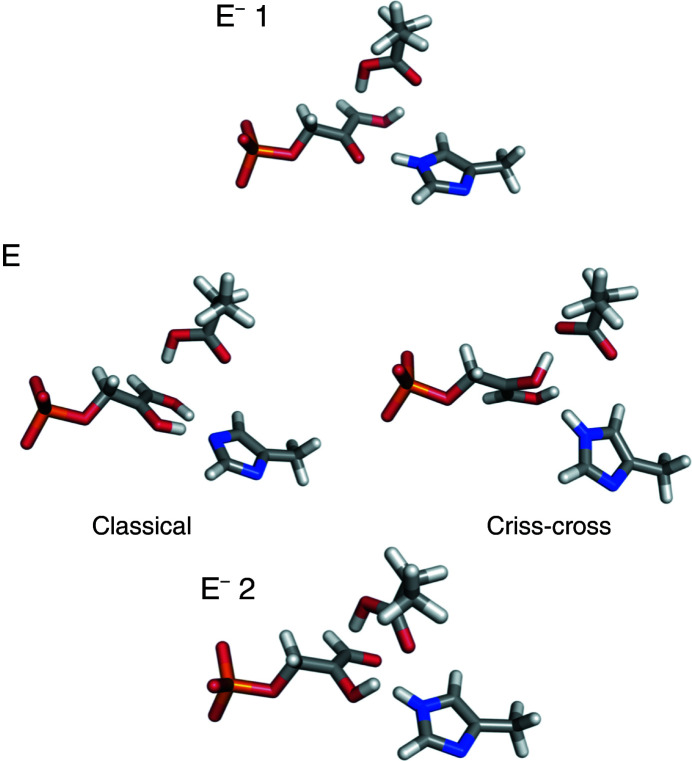
The three intermediates in the mechanism of TIM isomerization. E^−^ 1, enediolate intermediate 1; E, enediol intermediate; E^–^ 2, enediolate intermediate 2. The shuffle mechanism has no enediol intermediate.

The geometries of the reaction intermediates are shown in Fig. 9 ▸. Interestingly, the refined neutron structure of PGA–TIM is closest to the first enediolate intermediate, with an r.m.s.d. of only 0.29 Å, but the refined structure of PGH–TIM is also closest to this enediolate intermediate, albeit with a slightly higher r.m.s.d. of 0.33 Å. It should also be noted that the crystal structures of PGH–TIM and PGA–TIM are more similar to the structures of the intermediates from the QM/MM calculations than to the end-state structures. This shows that both of these inhibitors are suitable mimics of structures that characterize the intermediate steps in the TIM isomerization reaction, in line with the understanding that these tight-binding compounds can also be considered as transition-state analogues (Schramm, 2018 ▸).

## 4. Discussion   

Understanding enzymatic reaction mechanisms requires the interpretation and integration of results from several complex experimental and computational methods. While experimental methods such as X-ray and neutron crystallography, NMR, kinetics or other biochemical methods often provide individual data points or snapshots of an enzymatic reaction, computational chemistry methods are essential in tying together the experimental information and putting it into context. Yet, it is difficult to have confidence in even the most advanced computational methods if they do not reproduce the experimentally determined structures. Despite TIM being among the best studied enzymes both experimentally and computationally, not least because the reaction is rather simple, there has not been a consensus about its chemical mechanism. In particular, the controversy regarding the classical and criss-cross mechanisms has remained unresolved for decades.

The majority of atomic resolution structural information available comes from X-ray crystallography, which unfortunately lacks information about most hydrogen positions. While NMR can provide information about protonation states, a full picture of hydrogen positions, including the water molecules, in an enzyme active site usually requires neutron crystallography. As the reaction catalyzed by TIM mostly consists of movements of H atoms, the differences between the various mechanistic proposals are difficult to probe experimentally without structural information on H atoms. Even though neutron crystallography remains technically challenging, it has the power to resolve questions such as the nature of the general base catalyst in TIM. While early NMR data (Harris *et al.*, 1997 ▸) hinted at Glu167 being the general base, the neutron structure presented in this study unambiguously shows the protonation state of the glutamate. In the overall understanding of the catalytic mechanism of TIM, however, even such findings remain individual data points.

The more significant benefit of the neutron structures comes from the combination of experimental and computational methods, both by using the quantum-refinement approach to have better confidence in the structures and by using the inhibitor structures to study the real reaction computationally. It is important to realize that the crystallographic experimental data, the structure factors extracted from the diffraction images, also require prior information and somewhat subjective procedures to yield atomic coordinates. Therefore, the use of quantum chemistry to interpret the nuclear scattering-length density maps is essentially just adding another source of prior information for the structure determination. In the PGH–TIM complex, the hydroxylamine oxygen is rather disordered even in the atomic resolution X-ray structure, so the neutron structure understandably provides little information on the hydrogen bound to it. Yet, the quantum refinement allows us to build a model that is consistent with experimental data and the QM calculations, and hence is a chemically sensible model. The experimental information about the other parts of the structure is still valuable because it provides a well defined starting point for computational studies.

There are also limitations to the credibility of state-of-the-art computational calculations, mainly due to the assumptions on protonation states, water positions *etc.* that have to be made in the absence of structural information. In this case, the neutron structures of the inhibitor complexes resolve these ambiguities, so a computational method that reproduces these structures can be considered reasonably credible even for the substrate or product complexes, for which the overall structure is not very different. This provides a unique opportunity to computationally test three mechanisms without having a prohibitively large number of ambiguities in the protonation states. Therefore, the purely computational result that all mechanisms give a reasonable energetic path becomes much more credible than a similar result had been in the absence of the neutron structures.

## 5. Conclusions   

Our neutron structures and computational work have deepened the understanding of triosephosphate isomerase catalysis in several ways. Firstly, we have shown that the general base is definitely Glu167. Secondly, we have shown that there is no indication of any low-barrier hydrogen bonds. Thirdly, we show that the three suggested mechanisms are all energetically possible.

## 6. Abbreviations   

B3LYP, Becke, three-parameter, Lee–Yang–Parr functional.

CL, carbon link atom.

def2-SV(P), split-valence basis set with polarization functions on heavy atoms.

def2-TZVPD, valence triple-zeta basis set with polarization and diffuse functions on all atoms.

DFT-D3, dispersion-corrected density-functional theory.

DHAP, dihydroxyacetone phosphate.

GAP, glyceraldehyde 3-phosphate.

HL, hydrogen link atom.

LBHB, low-barrier hydrogen bond.

Lm, *Leishmania mexicana*.

MM, molecular mechanical.

NMR, nuclear magnetic resonance.

PGA, 2-phosphoglycolate.

PGH, phosphoglycolohydroxamate.

QM, quantum mechanical.

R.m.s.d., root-mean-square deviation.

TIM, triosephosphate isomerase.

TPSS, Tao–Perdew–Staroverov–Scuseria functional.

## Supplementary Material

PDB reference: triosephosphate isomerase, complex with 2-phosphoglycolate, X-ray, 7abx

PDB reference: joint neutron/X-ray, 7az3

PDB reference: joint neutron/X-ray, QM-refined, 7az4

PDB reference: complex with phospho­glycolohydroxamate, joint neutron/X-ray, 7az9

PDB reference: joint neutron/X-ray, QM-refined, 7aza

Supplementary Tables and Figures and explanations of the MTZ file column labels. DOI: 10.1107/S2052252521004619/fs5197sup1.pdf

MTZ and PDB files. DOI: 10.1107/S2052252521004619/fs5197sup2.zip

## Figures and Tables

**Table 1:** Refinement statistics of the refined (PDB entry 7az3) and quantum-refined (PDB entry 7az4) structures of PGA–TIM Geometry statistics were calculated with *MolProbity*. Values in parentheses are for the highest resolution shell.

	Refined	Quantum-refined
Resolution (X-ray) (Å)	25.33–1.137 (1.178–1.137)	25.33–1.137 (1.178–1.137)
Resolution (neutron) (Å)	30.39–1.70 (1.761–1.700)	30.39–1.70 (1.761–1.700)
*R*_work_ (X-ray)	0.1402 (0.2438)	0.1402 (0.2436)
*R*_free_ (X-ray)	0.1527 (0.2478)	0.1528 (0.2460)
*R*_work_ (neutron)	0.1819 (0.3234)	0.1821 (0.3233)
*R*_free_ (neutron)	0.2227 (0.3899)	0.2230 (0.3879)
R.m.s.d., bonds (Å)	0.013	0.013
R.m.s.d., angles (°)	1.1	1.1
Ramachandran favoured (%)	98.4	98.4
Ramachandran allowed (%)	1.6	1.6
No. of non-H atoms		
Total	2195	2195
Protein	1972	1972
Ligand	9	9
Solvent	214	214
Average *B* factors (Å^2^)		
Overall	26.0	26.1
Protein	24.6	24.8
Ligand	19.7	19.9
Solvent	38.6	38.7

**Table 2:** Refinement statistics of the refined (PDB entry 7az9) and quantum-refined (PDB entry 7aza) structures of PGH–TIM Geometry statistics were calculated with *MolProbity*. Values in parentheses are for the highest resolution shell.

	Refined	Quantum-refined
Resolution (X-ray) (Å)	45.05–1.095 (1.134–1.095)	45.05–1.095 (1.107–1.095)
Resolution (neutron) (Å)	30.55–1.800 (1.864–1.800)	30.55−1.80 (1.864–1.800)
*R*_work_ (X-ray)	0.1437 (0.3222)	0.1438 (0.3219)
*R*_free_ (X-ray)	0.1576 (0.3430)	0.1577 (0.3433)
*R*_work_ (neutron)	0.2201 (0.3708)	0.2202 (0.3705)
*R*_free_ (neutron)	0.2593 (0.3950)	0.2596 (0.3950)
R.m.s.d., bonds (Å)	0.013	0.013
R.m.s.d., angles (°)	1.5	1.2
Ramachandran favoured (%)	98.0	98.0
Ramachandran allowed (%)	2.0	2.0
No. of non-H atoms		
Total	2157	2157
Protein	1942	1942
Ligand	10	10
Solvent	205	205
Average *B* factors (Å^2^)		
Overall	22.6	22.8
Protein	21.2	21.4
Ligand	16.2	16.8
Solvent	35.8	36.0

**Table 3:** QM/MM relative energies (in kcal mol^−1^) at the TPSS-D3 and B3LYP-D3 levels (both with the def2-TZVPD basis set) of the intermediates in the TIM isomerization reaction in the classical, criss-cross and shuffle mechanisms

	Criss-cross		Classical		Shuffle	
	TPSSS	B3LYP	TPSS	B3LYP	TPSS	B3LYP
DHAP	0.0	0.0	0.0	0.0	0.0	0.0
TS1	9.7	6.4	9.7	6.4	9.7	6.4
Enediolate 1	9.4	5.4	9.4	5.4	9.4	5.4
TS2	15.2	13.3	17.1	13.6	—	—
Enediol	8.6	4.6	16.4	12.4	—	—
TS3	15.2	14.6	16.9	13.9	17.5	13.5
Enediolate 2	9.2	6.0	8.5	4.5	8.5	4.5
TS4	10.3	10.4	10.3	10.4	10.3	10.4
GAP	2.4	1.6	2.4	1.6	2.4	1.6
