# Supplementary material for: Neutron structures of Leishmania mexicana triosephosphate isomerase in complex with reaction-intermediate mimics shed light on the proton-shuttling steps
Source: IUCrJ. 2021 Jun 3;8(Pt 4):633–43. doi: 10.1107/S2052252521004619 (PMC8256706; doi:10.1107/S2052252521004619)
Supplement: Supplementary file 1 [file m-08-00633-sup1.pdf]

IUCrJ

Volume 8 (2021)

Supporting information for article:

Neutron structures of *Leishmania mexicana* triosephosphate isomerase in complex with reaction-intermediate mimics shed light on the proton-shuttling steps

Vinardas Kelpšas, Octav Caldararu, Matthew P. Blakeley, Nicolas Coquelle, Rikkert K. Wierenga, Ulf Ryde, Claes von Wachenfeldt and Esko Oksanen

Table S1 Data collection and processing

Values for the outer shell are given in parentheses.

Associated PDB	7AZ9, 7AZA		7AZ3, 7AZ4		7ABX
Inhibitor used	PGH	PGH	PGA	PGA	PGA
Diffraction source	P14 PETRA III	LADI-III ILL	BM30A ESRF	LADI-III ILL	BioMAX MAXIV
Wavelength (Å)	0.89	2.7-3.5	0.98	2.7-3.5	0.75
Temperature (K)	293	293	293	293	100
Detector	PILATUS 3	Neutron Image Plate	ADSC Q315	Neutron Image Plate	DECTRIS EIGER X 16M
Crystal-detector distance (mm)	146.9	200	110	200	158.53
Rotation range per image (°)	0.1	0	1	0	0.1
Total rotation range (°)	180	NA	180	NA	235
Exposure time per image (s)	0.04	21600	5	10800	0.01
Space group	C2	C2	C2	C2	C2
a, *b*, *c* (Å)	98.323, 52.678. 58.665	98.323, 52.678, 58.665	99.43, 52.89, 59.07	98.34, 52.37, 58.46	98.83, 52.92 58.66
α, β, γ (°)	90.00, 117.86, 90.00	90.00, 117.86, 90.00	90.00, 118.02. 90.00	90.00, 118.03, 90.00	90.00 118.45, 90.00
Resolution range (Å)	45.01-1.10 (1.16-1.10)	40.00-1.80 (1.90-1.80)	45.674-1.15 (1.18-1.15)	40.00-1.7 (1.79-1.7)	45.622-1.20 (1.27-1.2)
Total No. of reflections	337648	99141	495230	169169	364127

No. of unique reflections	104600	19145	95584	25565	83049
Completeness (%)	96.0 (88.88)	78.0 (60.)	99.4 (99.2)	88.6 (78.6)	99.2 (97.7)
Multiplicity	3.2 (2.9)	5.2 (4.1)	5.18 (2.9)	6.6 (6.8)	4.4 (4.4)
$\langle I/\sigma(I) \rangle$	14.77 (2.1)	3.8 (2.5)	23.49 (2.47)	13.3 (5.4)	6.41 (0.97)
R_{rmeas} %	4.3 (96.2)	19.4 (51.4)	4.9 (61.7)	9.7 (36.7)	11.5 (93.0)
CC(1/2)	99.9 (66.3)	95.5 (66.6)	99.9 (76.7)	99.7 (92.4)	99.4 (60.1)

Table S2 Refinement statistics of the refined (PDB ID 7ABX). Geometry statistics are calculated with MolProbity.

Highest resolution shell information is in parenthesis

Resolution (X-ray) (Å)	30.45-1.198 (1.241–1.198)
R_{work}	0.1489 (0.3091)
R_{free}	0.1805 (0.3349)
RMSD bonds (Å)	0.004
RMSD angles (º)	0.77
Ramachandran favoured (%)	97.98
Ramachandran allowed (%)	2.02
No. of non-H atoms	
Total	2414
Protein	1957
Ligand	9
Solvent	448
Average B-factors	
Overall (Å^2)	22.95
Protein (Å^2)	19.99
Ligand (Å^2)	18.56
Solvent (Å^2)	35.98

Column names found in the deposited reflection data:

F-obs-xray	The observed X-ray amplitudes
SIGF-obs-xray	The standard deviations of the observed X-ray amplitudes
R-free-flags-xray	The test set flags for cross validation
F-obs-neutron	The observed neutron amplitudes
SIGF-obs-neutron	The standard deviations of the observed neutron amplitudes
R-free-flags-neutron	The test set flags for cross validation
F-obs-filtered_xray	The observed X-ray amplitudes used in phenix.refine
SIGF-obs-filtered_xray	The standard deviations of the observed X-ray amplitudes used in phenix.refine
F-obs-filtered_neutron	The observed neutron amplitudes used in phenix.refine
F-model_xray	The X-ray amplitudes calculated from the model
PHIF-model_xray	The phases calculated from the model for the X-ray data
F-model_neutron	The neutron amplitudes calculated from the model
PHIF-model_neutron	The phases calculated from the model for the neutron data
2FOFCWT_xray	The $2mF_o - DF_c$ electron density map coefficients
PH2FOFCWT_xray	The $2mF_o - DF_c$ electron density map phases
2FOFCWT_no_fill_xray	The $2mF_o - DF_c$ electron density map coefficients without map filling
PH2FOFCWT_no_fill_xray	The $2mF_o - DF_c$ electron density map phases without map filling
FOFCWT_xray	The $mF_o - DF_c$ electron density map coefficients
PHFOFCWT_xray	The $mF_o - DF_c$ electron density map phases
2FOFCWT_neutron	The $2mF_o - DF_c$ nuclear scattering length density map coefficients
PH2FOFCWT_neutron	The $2mF_o - DF_c$ nuclear scattering length density map phases
2FOFCWT_no_fill_neutron	The $2mF_o - DF_c$ nuclear scattering length density map coefficients without map filling
PH2FOFCWT_no_fill_neutron	The $2mF_o - DF_c$ nuclear scattering length density map phases without map filling
FOFCWT_neutron	The $mF_o - DF_c$ nuclear scattering length density map coefficients
PHFOFCWT_neutron	The $mF_o - DF_c$ nuclear scattering length density map phases

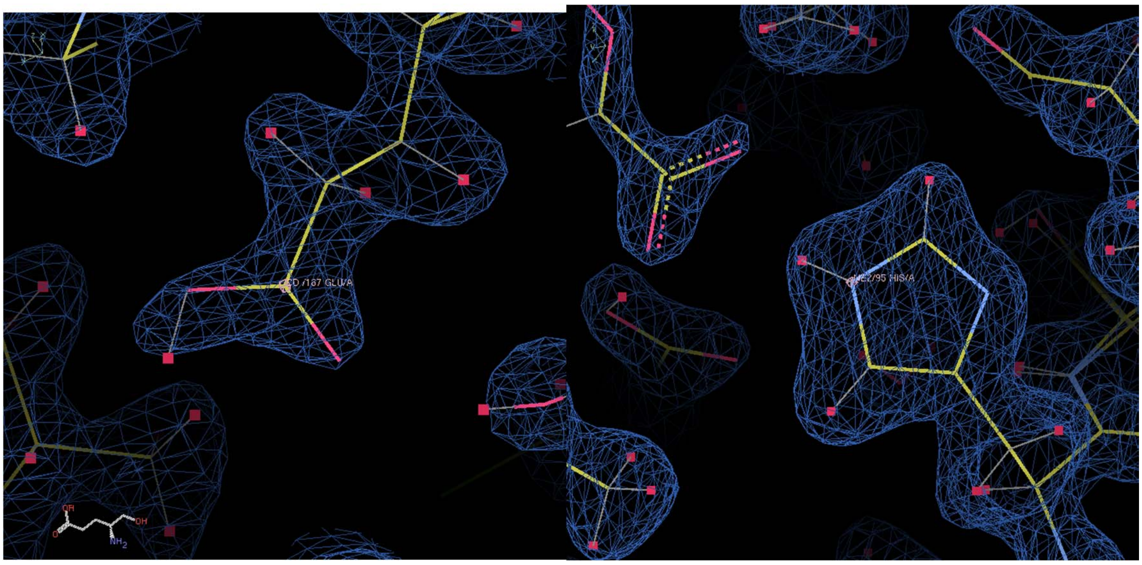

Figure S1 Nuclear density maps contoured at 1σ for the Glu-167 (left) and His-95 (right) residues in PGA-TIM, from Coot.

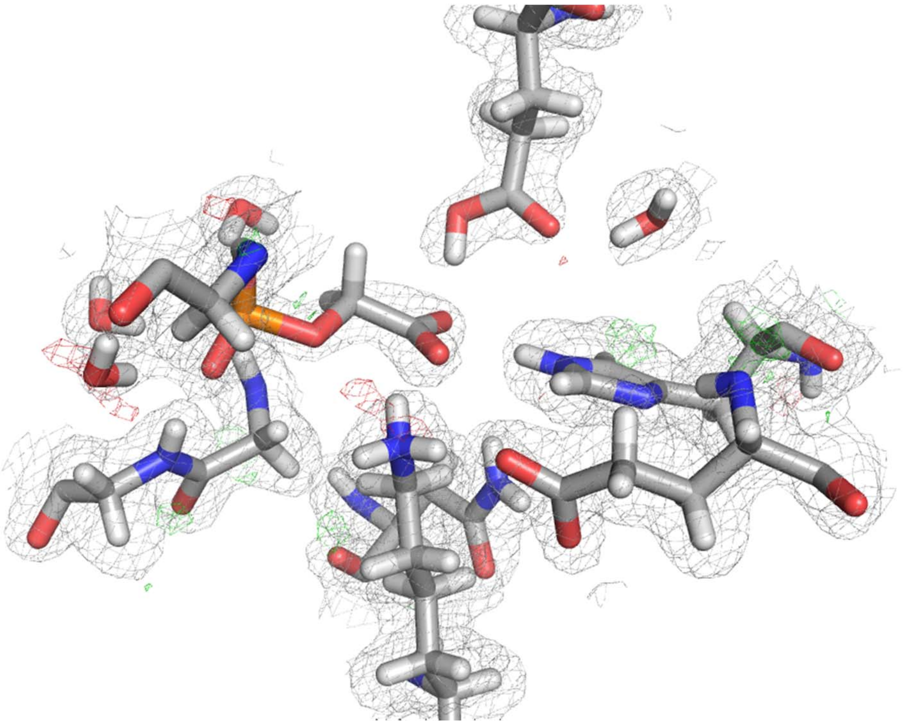

Figure S2 Joint X-ray–neutron refined structure of PGA–TIM active site and surrounding residues with the $2mF_o - DF_c$ nuclear scattering length density map contoured at 1.0 σ and the $mF_o - DF_c$ nuclear scattering length difference density maps contoured at 3.0 σ (green) and –3.0 σ (red).

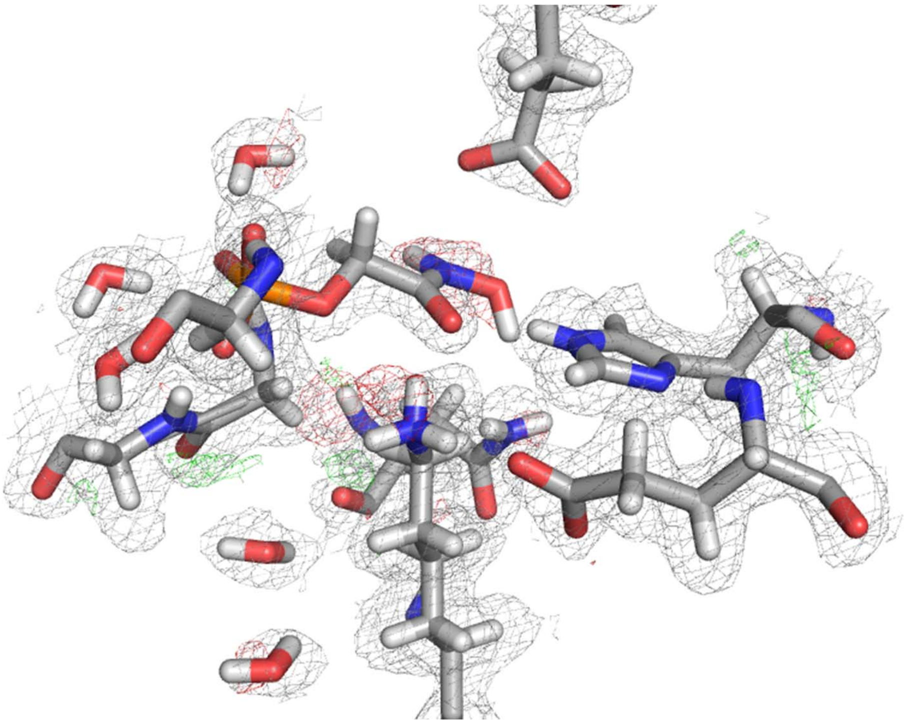

Figure S3 Joint X-ray–neutron refined structure of PGH–TIM active site and surrounding residues with the $2mF_o - DF_c$ nuclear scattering length density map contoured at 1.0 σ and the $mF_o - DF_c$ nuclear scattering length difference density maps contoured at 3.0 σ (green) and –3.0 σ (red).
